# A comparison of three-port and four-port Da Vinci robot-assisted thoracoscopic surgery for lung cancer: a retrospective study

**DOI:** 10.1186/s13019-024-02920-7

**Published:** 2024-06-26

**Authors:** Wenjian Jin, Liang Zheng, Xiao Fan, Hui Wang, Qianyun Wang, Chen Yang

**Affiliations:** 1 Department of Thoracic Surgery, The First People’s Hospital of Changzhou, The Third Affiliated Hospital of Soochow University, Changzhou, 213003 Jiangsu China; 2 Department of Hepatopancreatobiliary Surgery, The First People’s Hospital of Changzhou, The Third Affiliated Hospital of Soochow University, Changzhou, 213003 Jiangsu China; 3 Department of Pathology, The First People’s Hospital of Changzhou, The Third Affiliated Hospital of Soochow University, Changzhou, 213003 Jiangsu China

**Keywords:** Robot-assisted thoracoscopic surgery, Three port, Four port, Non-small cell lung cancer, Retrospective study

## Abstract

**Background:**

At present, research comparing the short-term postoperative outcomes of anatomical resection in lung cancer under different ports of da Vinci robot-assisted surgery is insufficient. This report aimed to compare the outcomes of three-port and four-port da Vinci robot-assisted thoracoscopic surgery for radical dissection of lung cancer.

**Methods:**

171 consecutive patients who presented to our hospital from January 2020 to October 2021 with non-small cell lung cancer and treated with da Vinci robot-assisted thoracoscopic surgery for radical resection of lung cancer were retrospectively collected and divided into the three-port group (*n* = 97) and the four-port group (*n* = 74). The general clinical data, perioperative data and life quality were individually compared between the two groups.

**Results:**

All the 171 patients successfully underwent surgeries. Compared to the four-port group, the three-port group had comparable baseline characteristics in terms of age, sex, tumor location, tumor size, history of chronic disease, pathological type, and pathological staging. The three-port group also had shorter operation time, less intraoperative blood loss, lower chest tube drainage volume, shorter postoperative hospitalization stay durations, but showed no statistically significant difference (*P* > 0.05). Postoperative 24, 48 and 72 h visual analogue scale pain scores were lower in the three-port group (*p* < 0.001). No significant difference was observed between the two groups in the hospitalization costs (*P* = 0.664), number or stations of total lymph node dissected (*p* > 0.05) and postoperative respiratory complications (*P* > 0.05).

**Conclusions:**

The three-port robot-assisted thoracoscopic surgery is safe and effective and took better outcomes than the four-port robot-assisted thoracoscopic surgery in non-small cell lung cancer.

## Introduction

Lung cancer has been reported to have the highest morbidity and mortality rates among all malignant tumors worldwide [1]. Surgery is the main treatment for resectable non-small cell lung cancer (NSCLC) [2]. With the development of surgical techniques, radical resection of lung cancer has evolved from open surgery to minimally invasive surgical approach. The first report of da Vinci robot-assisted thoracoscopic surgery was published in 2002 [3], and introduced to mainland China in 2009 [4]. Compared with open surgery, anatomic resections were done in da Vinci robot-assisted thoracoscopic surgery (RATS) through small incisions rather than rib spreading, which demonstrated RATS has a shorter postoperative hospital stay durations, lower postoperative complication rates and less postoperative pain [5, 6]. Compared with video-assisted thoracoscopic surgery (VATS), no significant difference was observed between two techniques in 30-day mortality [7, 8], intraoperative blood loss and operative times [9, 10], but RATS has the advantage of 3-D visualization, improved maneuverability and ergonomics such as wristed movements and instrument stability, which allows it to have more retrieved lymph nodes and nodal stations [11], less chest tube drainage, shorter chest tube duration and shorter postoperative length of stay [4]. These advantages have promoted the increasing use of RATS as an alternative to VATS [11]. Recently, the number and the size of surgical incision have been frequently considered, resulting in bi-port [12] and uni-port VATS [13]. At the same time, there is no unified standard for incision design and strategy in robot-assisted thoracoscopic lobectomy. At present, RATS usually requires 3–4 arms and multi-port pattern, such as 4–5 ports, for radical lung cancer [6, 14, 15]. Nevertheless, the general opinion is that fewer ports are better and help to reduce the amount of postoperative pain. Therefore, the ever-growing aesthetic demands and desire of satisfactory cosmesis make fewer surgical incisions imperative for thoracic surgeons. Based on this consideration, the transition from the four-port approach to the three-port approach has occurred in our department. The difference between two methods lies in the location and number of surgical incisions. However, there are no reports comparing the clinical efficacy of two methods. This retrospective study aimed to compare the clinical outcomes of three-port and four-port da Vinci robot-assisted thoracoscopic surgery for non-small cell lung cancer and facilidate the adoption of three-port strategy.

## Methods

### Study design and data collection

This study is a retrospective comparative study performed in a single center and was conducted in accordance with the Declaration of Helsinki (as revised in 2013). This study was approved by the Ethics Committee of the Third Affiliated Hospital of Soochow University (Approval No. 2021 technology 97) and informed consent was taken from all the patients. All the patients gave written informed consent to participate in the research. The indications and contraindications for robotic lobectomy and segmentectomy were comparable to those reported previously for VATS [16]. Some conditions such as thoracic dense adhesion, advanced disease, neoadjuvant radiotherapy and hilar-dense nodal invasion were regarded as relative contraindications.

### Patient selection and grouping

**Fig. 1:**
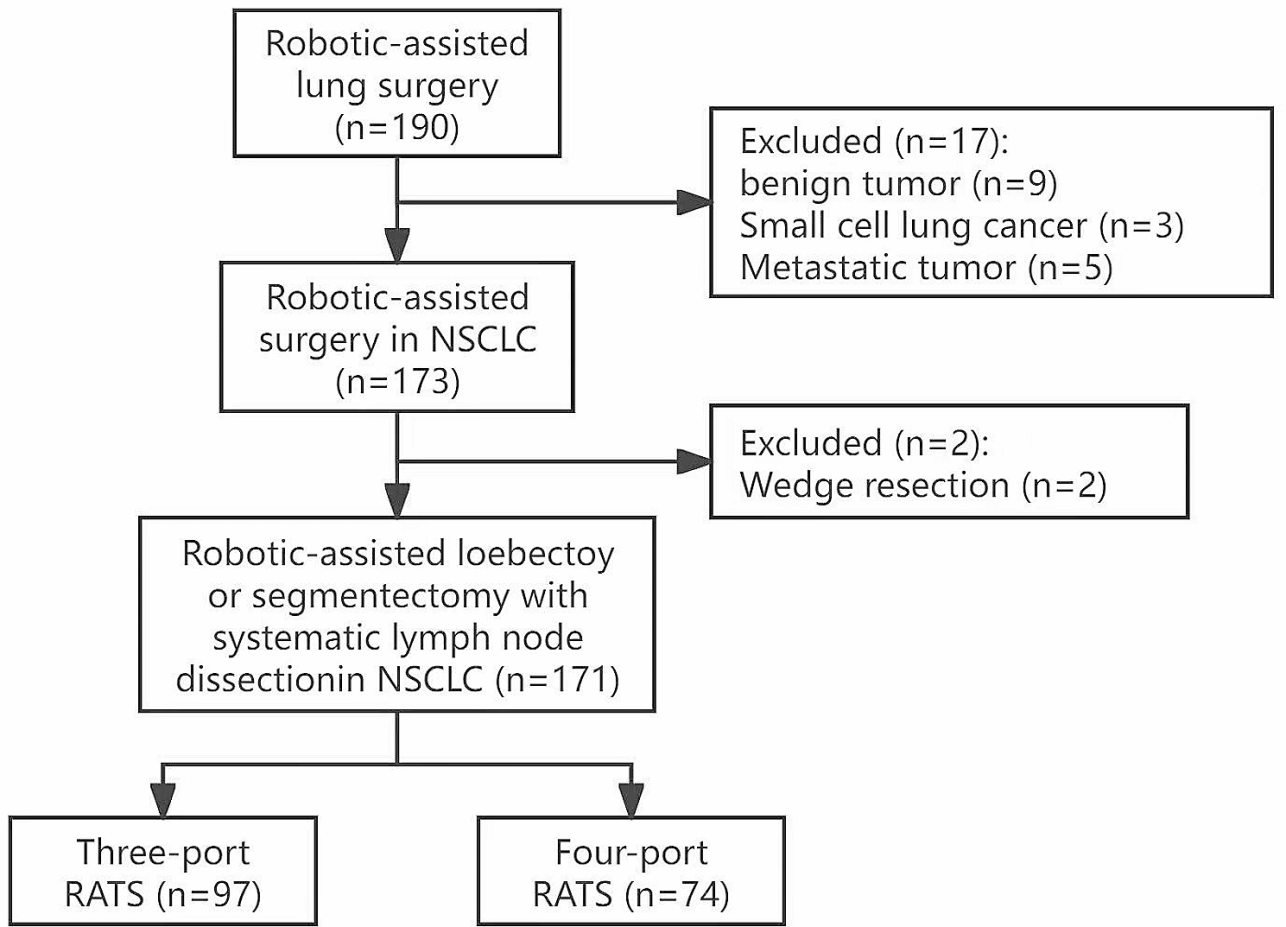
Schema of patient selection

As shown in Fig. 1, from January 2020 to October 2021, 190 patients with NSCLC were treated at a single institution. Among them, 9 cases diagnosed as benign tumors, 5 cases of metastatic tumor and 3 cases of small cell lung cancer (SCLC) were excluded. Additionally, 2 cases of wedge resections were excluded. Consequently, 171 patients pathologically diagnosed with NSCLC were included in this study and were divided into the three-port and four-port groups, including 97 cases in the three-port group and 74 cases in the four-port group. Patients diagnosed with lung cancer admitted to the department of thoracic surgery were enrolled in our study in case of meeting the criteria we made. Data were collected retrospectively. The status of comorbidity was objectively assessed using the Charlson comorbidity index (CCI), which was developed in 1987 [17] and originally included 19 medical conditions. In our study, we used the revised version of CCI encompassing 23 medical conditions (Table 1), which was found to better predict health outcomes comparing with the original version. Actually, robotic-assisted thoracic surgery in our group with the three-arm, four-port approach was first performed in May 2019, and in June 2020 the three-arm, three-port approach was just started. Three robotic arms and one utility incision in the four-port approach were 8–10 cm away from each other to avoid arm impingement and interference, this advantage made it acceptable for novices experienced in VATS surgery [18]. The three-port approach is currently the standard procedure in our department with few intraoperative conversion to four-port approach despite of thoracic dense adhesion, or severe calcification of hilar lymph nodes. However, the enrolled patients were all collected after the 50th cases respectively to eliminate the technical bias, which was far beyond the requirements of the average learning curve of 20 cases [19, 20].

**Table 1:** CCI and prevalence of comorbid conditions of all patients (*n* = 171)

Score	Condition	Number of patients (%)
1	Coronary artery disease/ myocardial infarction	3(1.8%)
	Congestive heart failure	
	COPD/Asthma	1(0.6%)
	Hypertension	33(19.3%)
	Peripheral vascular disease	
	Mild liver disease	1(0.6%)
	Cerebrovascular disease	
	Connective tissue disease	
	Diabetes without end organ damage	9(5.3%)
	Dementia	
	Depression	
	Ulcer disease	
	Takes warfarin	1(0.6%)
2	Hemiplegia	
	Moderate to severe renal disease	
	Diabetes with end organ damage	
	Any prior tumor	
	Skin ulcers/cellulitis	
	Leukemia	
	Lymphoma	
3	Moderate to severe liver disease	
6	Metastatic solid tumor	
	HIV/AIDS	

CCI: Charlson comorbidity index

The inclusion criteria were based on both preoperative and intra-operative evaluation as follows: (1) each patient underwent radical lung cancer using three-port RATS or four-port RATS. (2) clinical staging of T_1 − 3_N_0 − 1_M_0_. (3) histopathologically proven NSCLC. (4) no distant metastasis (5) no neoadjuvant Therapy. (6) Patients who can tolerate surgery.

The exclusion criteria based on both preoperative and intra-operative evaluation were as follows: (1) palliative resection. (2) Patients who had any contraindication for RATS. (3) Patients who had a history of thoracic surgery. (4) Patients who did not give consent. Patients were also excluded if the number and stations of dissecting lymph nodes did not meet the criteria of what IASLC proposed [21].

All operations were performed by a highly experienced surgeon. According to the surgical method, the included patients were categorized into the three-port group (*n* = 97) and four-port group (*n* = 74).

The patient’s preoperative examinations include: routine blood tests, coagulation, immunohistochemistry, blood biochemistry, electrocardiograms, echocardiography, pulmonary function tests, fiberoptic bronchoscopy, chest computed tomography (CT). Brain magnetic resonance imaging (MRI), bone scintigraphy, abdominal and bilateral adrenal ultrasonography to exclude distant metastasis. Some patients underwent positron emission tomography/computed tomography (PET/CT) if necessary. Endobronchial ultrasound-guided transbronchial needle aspiration was used preoperatively in patients with lymph nodes suspicious of malignancy (on CT or PET-CT with FDG uptake in the nodes), the same method was did even if meeting a central tumor or a tumor larger than 4 cm, without suspecting malignancy of lymph nodes on imaging. Mediastinoscopy was not performed in this study. TNM staging was based on the eighth edition of the International Association for Lung Cancer Research (IASLC) guidelines. Postoperative complications were evaluated by the Clavien-Dindo classification [22, 23]. Clavien-Dindo grade 1–2 complications were classified as minor complications, and Clavien-Dindo grades 3–5 complications were classified as major complications. The intensity of postoperative pain was scored in the first 24, 48 and 72 postoperative hours with the visual analogue score (VAS) [24, 25]. The scale is an integer scale of 0–10, where 0 is no pain and 10 is the worst imaginable pain.

### Surgical procedures

Patients were placed in the lateral decubitus position with the operating table flexed to increase the intercostal spacing, and the anesthesiologist gave the patient general anesthesia. Then a double lumen endotracheal tube was used to achieve single lung ventilation. Pulmonary resection was defined as lobectomy/ segmentectomy following with lymphadenectomy, which included the dissection of an entire lobe or removal of lung segment and individual interruption of the target pulmonary artery, vein, and lobar or segmental bronchus, as well as radical lymph node dissection. All surgeries were performed with the da Vinci Xi system, which was positioned at the patient’s head and left side. Before the pulmonary segment dissection was performed, 3D computed tomography bronchography and angiography (3D-CTBA) was used to help to identify the involved vessels and bronchus and the expansion collapse method was used to recognize the boundary with the normal lung tissue. The specimen was dissected using the energy equipment and the Endo-GIA staplers, and subjected to intraoperative frozen section diagnosis, and the malignant reports guided the systematic lymph node dissection. One 28 Fr chest tube and one silicon sphere were placed respectively after surgery.

**Fig. 2:**
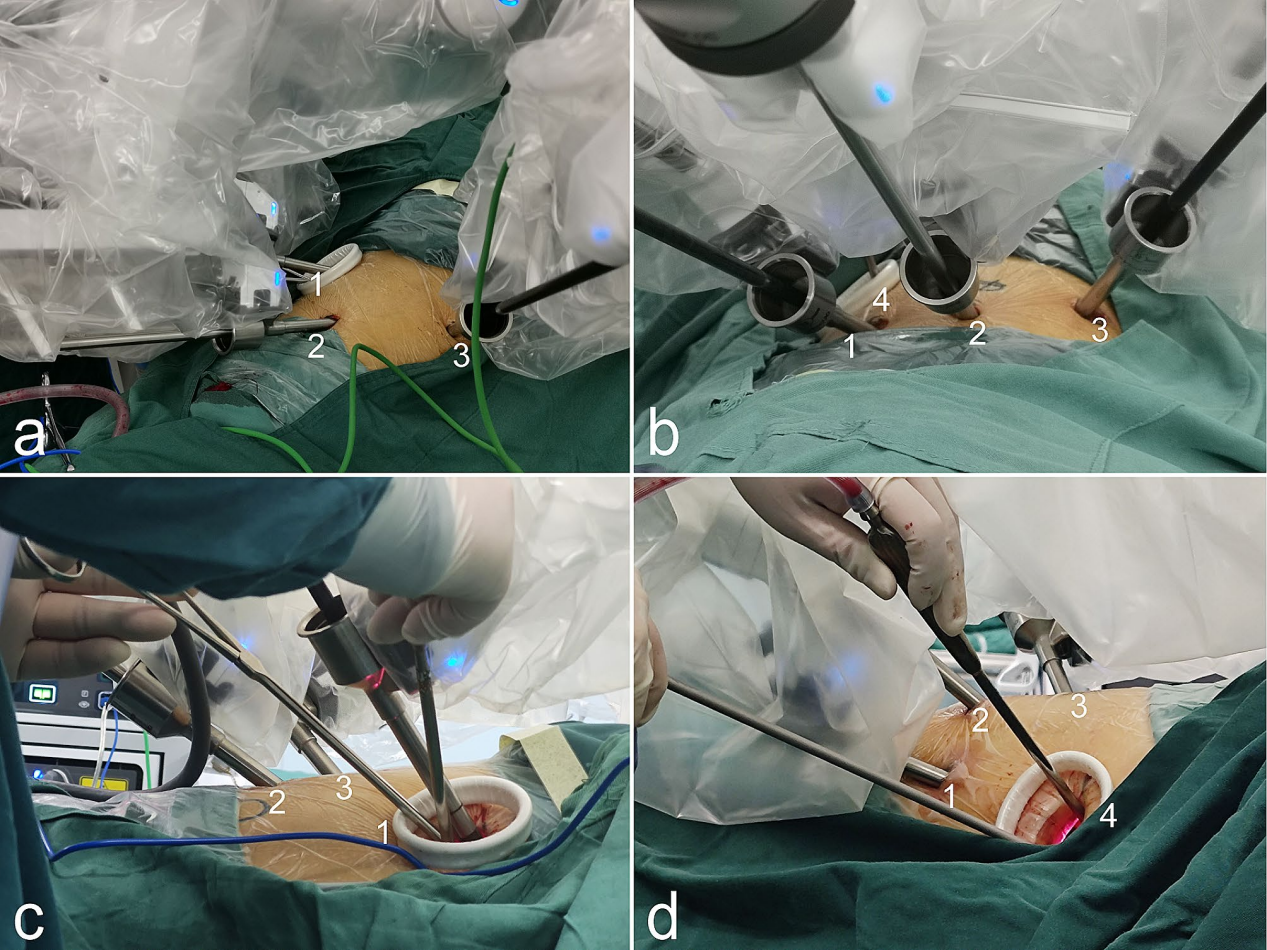
Port placements in the three-port group and four-port group. (**a**, **b**) patient in a right lateral decubitus position and port placement in the three-port and four-port group: the robotic arm 1, equipped with Maryland bipolar forceps (surgeon left hand), the robotic arm 2, equipped with 30-degree-angle-down stereoscopic camera, the robotic arm 3, equipped with permanent cautery hook (surgeon right hand). The bedside assistant and robotic arm 1 share the same incision in the three-port group, while the 3 cm utility incision as port 4 was used only by the bedside assistant in the four-port group. (**c**, **d**) The equipments in robotic arms 1, 3 were as opposed to figure a, b, as the two robotic arms were held by opposite hands. The robotic arm 2 was still equipped with stereoscopic camera

Three-port group: Fig. 2a, c showed the port placement of the three-port approach. A 8 mm camera port incision was made at the eighth intercostal space midaxillary line, and a 30-degree three dimensional camera arm was placed to provide a field of view for placing two other instrument arms. Then a 3 cm utility incision was made at the fifth or sixth interspace on the anterior axillary line, which was used by the bedside assistant and a robotic arm after placing the trocar sleeve. Finally a 8 mm incision was made at the eighth intercostal space infrascapular line (Fig. 2).

Four-port group: Fig. 2b, d showed the port placement of the three-port approach. The anesthesia was the same as in the three-port group. A 8 mm camera port incision was made at the eighth intercostal space midaxillary line, and a 30°camera arm was placed to provide a field of view for placing three other instrument arms. Then a 3 cm utility incision was made at the fifth interspace on the anterior axillary line. One 8 mm incision was made at the eighth intercostal space infrascapular line and the other 8 mm incision was symmetrically created in the seventh intercostal space between the anterior axillary line and mid-axillary line, these two incisions were used for the trocars, which were the robotic arm working channel. After the trocar sleeve was placed, the utility incision was used by the bedside assistant to assist surgeons (Fig. 2).

The management of postoperative pain depended on continuous analgesic pump system, which was inserted through the port between the intrapleural space covering the multi-level intercostal area. After the operation, oral pain killers such as NSAID drugs or tramadol were initiated at postoperative day 1. All patients were treated with subcutaneous injection of low-molecular-weight heparin to achieve antithrombotic prophylaxis if the drainage fuid was not bright red, which was continued until discharge.

### Statistical analysis

The Shapiro-Wilk’s test was performed to verify the normal distributions of continuous variables. Continuous variables were presented as mean and standard deviation and compared by Student’s t-test in case normal distributions were verified. Continuous variables that were not normally distribution were expressed as the median (interquartile range) and compared between groups with the Mann-Whitney U-test. Categorical variables were presented as frequency and percentage and compared by the Chi-square and Fisher’s exact test. Since the distribution of age, gender, smoking history, pulmonary function, tumor size and clinical stages were comparable between the 2 groups, propensity score matching was not performed in further analysis. SPSS software was applied for data analysis, and a statistically significant difference was considered for a value of *P* < 0.05.

## Results

### Homogeneity of patients

There were 171 patients who underwent robot-assisted lobectomy/ segmentectomy and lymphadenectomy successfully from January 2020 to October 2021 with no conversion to open surgery or 30-day mortalities. Among them, 63 were men and 108 were women; there were 157 cases of adenocarcinoma and 14 cases of squamous cell carcinoma. There were 98 stage IA cases, 28 stage IIA cases, 22 stage IIB cases and 23 stage IIIA cases. All patients were divided into the three-port group (*n* = 97) and the four-port group (*n* = 74). No significant difference was observed between the two groups in sex (*P* = 0.404), age (*P* = 0.811), forced expiratory volume in 1 s (FEV1) (*P* = 0.317), smoking history (*P* = 0.562), American Society of Anesthesiologists risk class (*P* = 0.891), resection of entire lobe or segment (*P* = 0.846), tumor size (*P* = 0.647), tumor location (*P* = 0.402) and CCI (*p* = 0.462) (Table 2). The prevalences of comorbid conditions of all patients are summarized in Table 1. The most common comorbid conditions were hypertension and diabetes, followed by coronary artery disease.

**Table 2:** Baseline clinical characteristics of the study subjects

Variables	Three-port group (*n* = 97)	Four-port group (*n* = 74)	*p*-Value
Age (years)	62 (58–69)	64 (60–71)	0.811
Sex			0.404
Male	35 (36.1%)	28 (37.8%)	
Female	62 (63.9%)	46 (62.2%)	
Smoking history			0.562
Yes	15 (15.5%)	10 (13.5%)	
No	82 (84.5%)	64 (86.5%)	
FEV1% predicted	97.6 (91.6–102.3)	95.8 (89.4–99.7)	0.317
American Society of Anesthesiologists risk class			0.891
I	5 (5.2%)	4 (5.4%)	
II	80 (82.3%)	60 (81.1%)	
III	12 (12.5%)	10 (13.5%)	
IV	0 (0%)	0 (0%)	
V	0 (0%)	0 (0%)	
Tumor size (cm)	1.92 ± 1.23	1.88 ± 1.04	0.647
Tumor location			0.402
left upper lobe	29 (29.8%)	19 (25.7%)	
left lower lobe	15 (15.5%)	9 (12.2%)	
right upper lobe	31 (32.0%)	28 (37.8%)	
right middle lobe	5 (5.2%)	5 (6.8%)	
right lower lobe	17 (17.5%)	13 (17.5%)	
Lobectomy/ Segmentectomy			0.846
Lobectomy	76 (78.4%)	45 (60.8%)	
Segmentectomy	21 (21.6%)	29 (39.2%)	
Charlson comorbidity index	2.825	2.653	0.462

### Operation overview

**Fig. 3:**
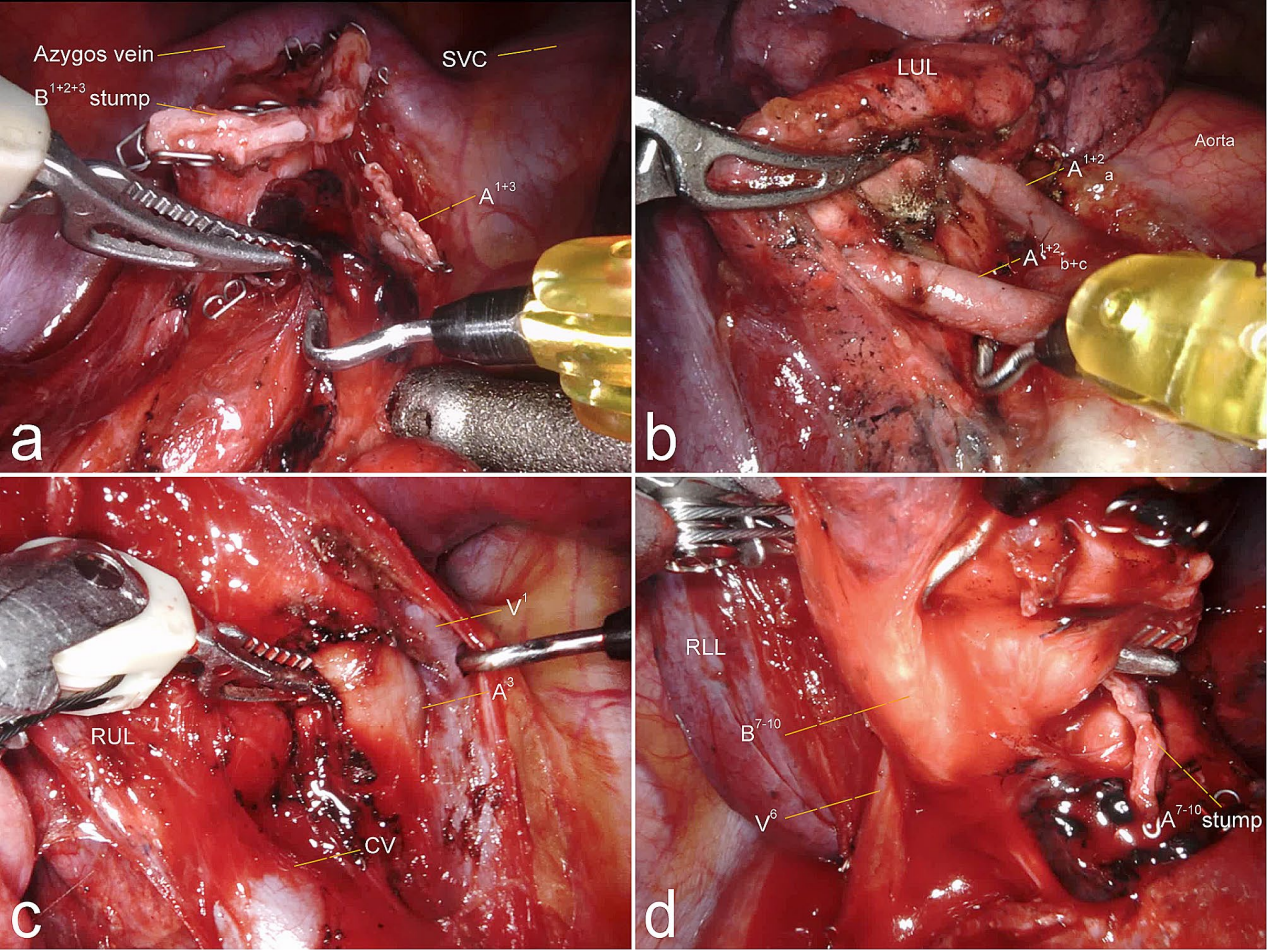
Intraoperative views. **a**: Hilar lymph nodes were dissected during robotic-assisted right upper lobectomy; **b**, A^1 + 2^a and A^1 + 2^b + c were clearly defined during robotic-assisted left upper division segmentectomy (LS^1 + 2^+S^3^); **c**, A^3^ was confirmed and Maryland bipolar forceps passed though it creating a tunnel with the cooperation of cautery hook during robotic-assisted right anterior segmentectomy (RS^3^); **d**, Maryland bipolar forceps passed though B^7–10^ creating a tunnel for staplers during robotic-assisted right basilar segmentectomy(RS^7–10^) LUL: Left upper lobe; RUL: Right upper lobe; RLL: Right lower lobe; SVC: superior vena cava; CV: central vein

The operative features were shown in Fig. 3; Table 3. There was no conversion from minimally invasive surgery to thoracotomy. The intraoperative blood loss was less in the three-port group than in the four-port group, but showed no significance (*P* = 0.406). Meanwhile, there was no significant difference in operative time (*P* = 0.314), number of lymph nodes retrieved (*P* = 0.715 and *P* = 0.637) or nodal stations explored (*P* = 0.917 and *P* = 0.955).

**Table 3:** Comparison of perioperative parameters between the two groups

Variables	Three-port group (*n* = 97)	Four-port group (*n* = 74)	*p*-Value
Operative time (min)	98 (83–118)	108 (92–129)	0.314
Intraoperative blood loss (ml)	96 (82–114)	108 (89–127)	0.406
Chest tube duration (days)	3 (2–5)	4 (2–6)	0.206
Postoperative thoracic drainage (ml)	474 (392–585)	724 (395–790)	0.084
Postoperative hospital stay (days)	4 (3–6)	5 (3–8)	0.114
Pathological types			0.603
Adenocarcinoma	87 (89.7%)	70 (94.6%)	
Squamous cell Carcinoma	10 (10.3%)	4 (5.4%)	
Total number of lymph nodes dissected			
N1	6.82 ± 2.38	6.95 ± 1.94	0.715
N2	5.86 ± 2.15	6.21 ± 2.02	0.637
Total number of lymph node stations dissected			
N1	3.09 ± 1.12	3.16 ± 0.68	0.917
N2	3.54 ± 1.16	3.71 ± 1.03	0.955
pTNM stage			0.241
IA1	20 (20.6%)	16 (21.7%)	
IA2	22 (22.7%)	20 (27.0%)	
IA3	10(10.3%)	10 (13.5%)	
IIA	18 (18.6%)	10 (13.5%)	
IIB	14 (14.4%)	8 (10.8%)	
IIIA	13 (13.4%)	10 (13,5%)	
24 h postoperative VAS pain scores	4.55 ± 0.61	5.31 ± 0.84	<0.001
48 h postoperative VAS pain scores	3.21 ± 0.58	3.99 ± 0.49	<0.001
72 h postoperative VAS pain scores	2.11 ± 0.41	2.62 ± 0.68	<0.001
hospitalization costs (CNY)	72263.46 ± 7865.37	74622.83 ± 8451.48	0.664

### Postoperative recovery condition

The number of postoperative days in the three-port group before chest tubes were removed was less than that in the four-port group (3.34 ± 0.93 vs. 3.65 ± 1.20 days, respectively, *p* = 0. 206). There was no significant difference in terms of chest tube drainage volume (*P* = 0.084), postoperative hospitalization (*P* = 0.114) and hospitalization costs (*P* = 0.664) between the two groups. The similar result could be observed in the type of histological classification (*P* = 0.603) and pathological stage (*P* = 0.241), which was detailed in Table 3. The postoperative 24, 48 and 72 h visual analogue scale (VAS) pain scores were significantly lower in the three-port group than in the four-port group (all *P* < 0.001), which showed the three-port approach had more benefit in postoperative pain. Postoperative complications in the two groups are presented in Table 4. Each group had one patient suffered from pulmonary infection, atelectasis and pneumothorax. The patient who suffered severe cough and aggravated air leakage at the same time developed into obvious subcutaneous emphysema. The most common complication was pulmonary air leakage, which might be dictated by stapler fault and postoperative severe cough. Chylothorax was observed in one patient in the four-port group, but was not observed in any patient in the three-port group. There was no significant difference in the occurrence rate of complications in both groups (all *P* > 0.05). All these patients recovered with conservative treatments without requiring reoperation.

**Table 4:** Postoperative morbidity

Variables	Three-port group (*n* = 97)	Four-port group (*n* = 74)	*p*-Value
Minor complications (Clavien-Dindo grades 1–2)			
Pulmonary infection	1 (1.0%)	1 (1.4%)	0.477
Pulmonary air leakage Atelectasis	3 (3.1%) 1 (1.0%)	4 (5.4%) 1 (1.4%)	1.105 1.642
Arrhythmia	1 (1.0%)	0 (0%)	0.524
Major complications (Clavien-Dindo grade 3–5)			
Chylothorax	0 (0%)	1 (1.4%)	0.873
Pulmonary embolus	1 (1.0%)	0 (0%)	1.052
Obvious subcutaneous emphysema or pneumothorax	1 (1.0%)	1 (1.4%)	1.603
Required reoperation for bleeding	0 (0%)	1 (1.4%)	1.025

## Discussion

Surgical techniques for pulmonary resection are constantly being developed. In recent years, RATS has been increasingly used as a safe and effective alternative to open surgery or VATS [26]. Compared with open procedures, RATS had the advantages of shorter length of hospital stay and lower postoperative pain scores [4, 5]. Likewise, compared with VATS, RATS is safer than VATS when considering its less intraoperative blood loss, shorter drainage times, shorter postoperative hospital stay durations and comparable conversion and re-operation rate for NSCLC [4]. These advantages have made RATS popular throughout China over the past decade. However, there is no unified standard for incision design and strategy for robot-assisted pulmonary resection. Reduction of postoperative pain and improvement of life quality can be obtained with the help of fewer incisions while lower cost can be achieved by using fewer arms [18]. Robotic-assisted pulmonary resection was performed during our early robotic surgery. Over this same time period, we used four-port method with three 0.8 cm incisions and a 3 cm additional incision for assistant surgeon, which was consistent with aforementioned report [27]. As we all know, three-port VATS has gained popularity and is now widely adopted by worldwide thoracic surgeons [28]. We usually perform thoracoscopic lobectomy using three port surgical approach. We believe the number of incisions in robotic-assisted pulmonary resection should not exceed those in VATS to preserve the advantages of minimal invasiveness. Considering patients’ sensitiveness to incision number and size, in 2020, we devised a newly three arm three port method without degrading the quality of RATS using the same incision of robotic arm 1 and assistant hole (Fig. 2), where the anterior trocar is placed at the upper end of the utility incision sleeve with 2 cm space left for assistant to help at the lower end. The space apart from the trocar was adequate for suction, retraction, palpation of the nodule and extraction of the specimen, such as the lobe, segment, or lymph node. Compared to four-port RATS, the three-port technique has the following advantages. First, the port mapping is similar to the conventional three-port VATS, which facilitates the transition and adaptation of thoracic surgeon in RATS, even those with inadequate training and experience. Second, if encountering an emergency situation, such as severe bleeding or malfunction of the robot, we can immediately remove the robotic arms and switch to VATS without the need for additional incisions. Third, the use of fewer incisions can be associated with reduced time and cost, reduced nerve damage and pain around the incision, also can avoid decrease scar formation with better cosmesis and ameliorate the postoperative quality of life.

To date, no studies have compared the outcomes of these two methods. In this study, we found that three-port RATS can achieve the comparable number or stations of total lymph node dissected without increasing the operation time or postoperative respiratory complications rate compared to four-port RATS, suggesting that both methods were equivalent in surgical effect. Although there was no significant difference between the two groups in terms of in terms of the intraoperative blood loss, chest tube drainage, drainage times and postoperative hospital stay durations, relative lower results represented that three-port approach was also an effective and safe method during RATS. The operation duration of the three-port group was not significantly prolonged even if complex segmentectomies were performed (Fig. 3), such as basilar segmentectomy or left upper division segmentectomy (S1 + 2 + S3), rather than simple segmentectomies, such as lingulectomy or dorsal segmentectomy. However, we cannot definitively state that the three-port method is superior to conventional four-port method. By contrast, we believe that the three-port method is not significantly inferior to the existing method.

Our data demonstrated that fewer surgical ports greatly reduced surgical trauma and hastened postoperative recovery. Table 4 showed that one patient in the four-port group underwent reoperation due to the bleeding of incision. The hole was missing in the three-port RATS, which was closed to the pericardium in the left cavity and the diaphragm in the right cavity. Iatrogenic injury with hematorrhea was easily induced even though the trocar was placed meticulously under the guidance of robotic camera. Therefore, we decided to omit this incision.

Postoperatively, pain adversely affects patients’ postoperative rehabilitation, daily activities and quality of life [29]. Postoperative pain relief is of positive significance for improving postoperative quality of life. The degree of pain alleviation became apparent with the administration of various drugs. If this was not sufficient, their oral dose of analgesics was increased and analgesic pump was started, which shows that RATS can still be a painful surgical procedure. Our study showed that the postoperative 24, 48 and 72 h VAS scores in the three-port group were significantly lower than those in the four-port group. As is well known, the incision was made in the 7th intercostal space, which was different from the camera port and port 3 in the 8th intercostal space. Reduction in intercostal nerve injury and neuropathic pain can be achieved by omitting this incision. And a wound protector is required to protect the incision from overstimulation of nerves by surgical instruments. Pain after surgery for lung cancer has received increasing attention over the past decade. The Numerical Rating Scales (NRSs), the Visual Analogue Scales (VASs), the Verbal Rating Scales (VRSs), and the Faces Pain Rating Scales (FPSs) are commonly used pain intensity scales. General opinion is that NRSs have more validity and more strengths than other scales [30], but more research is needed to further confirm this finding. Nevertheless, previous researches also revealed that the VAS, like the NRS, is a more “pure” measure of pain intensity, as a measure with less verbal cues than VRS or affect-related cues than FPS [31]. In our department, we have specifically designated a nurse to carry out VAS measurement, which is easy-to-use and does not need a sophisticated device, judging both severity of pain and the extent of pain relief. However, in the clinical practice, this tool still has significant limitations. The patients were required to draw a point consistent with the pain intensity in a straight line according to their painful severity, just this process need them to have adequate levels visual acuity and abstract thinking, considerable difficulty appearing especially in the elderly, populations with low degree of education and communication deficits. On the other hand, VAS was inappropriate to use in the emergency situation. Therefore, NRS may be a preferred scale in our future research.

As we all know, mediastinal nodal dissection plays a vital role in radical lung cancer surgery, affecting both pathological N staging and subsequent treatment strategies in addition to patient outcomes [32]. In our hospital, the standard pulmonary resection consists of lobectomy and systematic lymph node dissection, following the guidelines of lung cancer treatment. All cases in both groups met the criteria for complete resection. As for comparison of the three-port group and four-port group for radical dissection of lung cancer, in this study, there were no significant differences between the two groups in the number or stations of total lymph node dissected. In 2021, Huang et al. [33] reported the outcomes of 685 patients with stage I-IIIA who underwent robotic lobectomy. They found that the number of dissected lymph nodes was 14.87 ± 2.05 and stations were 6.19 ± 1.01 among these patients, which is similar to the results reported in the present study, indicating that lymph node dissection in our department was thorough enough. Once the surgical field was fully exposed with the help of robotic arms and bed assistant, fewer ports don’t limit the operating angle or increase the difficulty of lymph node dissection. In 2023, Anna et al. [34] compared the outcomes of 246 pulmonary resections with systematic lymph node dissection for clinical stages I–II NSCLC. The total number of dissected lymph nodes and stations was significantly higher in RATS. To improve a more visible operative field, we would choose to change the location of the utility incision. When the tumor was found in the left upper lung, we would choose the sixth intercostal space on the anterior axillary line as the utility incision instead of the fifth intercostal space when meeting other lobes.

The minor complication rates were similar in both the groups: one pulmonary infection, three pulmonary air leakage, one atelectasis and one arrhythmia in the three-port group (*n* = 97), one pulmonary infection, four pulmonary air leakage and one atelectasis in the four-port group (*n* = 74). All patients in the three-port group recovered with conservative treatments without requiring reoperation, while one patient who underwent reoperation in the four-port group developed bleeding of incision. No significant difference was observed between the two groups in chylothorax (*P* = 0.873), pulmonary embolus (*P* = 1.052), obvious subcutaneous emphysema or pneumothorax and required reoperation (*P* > 0.05) (Table 4). These results reveal that fewer ports did not affect the incidence of these postoperative complications with the similar extent of resection for tumors, which confirmed that the three-port group is equivalent to the safety of the four-pour group.

The limitations of this study are the retrospective design and small sample size. So it still needs to be further verified by large sample size randomized controlled trials. Whether the advantages of the three-port RATS can bring long-term survival benefits is not clear, we are looking forward to the comparison of long-term survival results.

In conclusion, three-port RATS is a safe and effective surgical procedure in the patients with early staged operable lung cancer, which has the advantages of reduction of postoperative pain and improvement of cosmetic results whereas unaffecting clinical outcomes. With the time going, we believe that the three-port technique will be a feasible alternative to the four-port technique and widely adapted by more thoracic surgeons worldwide.

## Data Availability

No datasets were generated or analysed during the current study.

## Abbreviations

CCI: Charlson comorbidity index
CT: Computed tomography
IASLC: International Association for Lung Cancer Research
MRI: Magnetic resonance imaging
NSCLC: Non-small cell lung cancer
PET/CT: Positron emission tomography/Computed tomography
RATS: Robot-assisted thoracoscopic surgery
VAS: Visual analogue score
VATS: Video-assisted thoracoscopic surgery
